# Deep Sedation in High-risk Patients Undergoing Emergency Upper GI Endoscopy: A Retrospective Study Assessing Safety and Effectiveness

**DOI:** 10.37825/2239-9747.1067

**Published:** 2024-12-26

**Authors:** Martina Mariani, Romolo Villani, Anna De Simone, Simona Cotena, Rossella Pirolli, Manuela Nugnes, Silvio Canciello, Paolino Manganiello, Elena Santoriello, Francesca Schettino, Vincenzo Schettini, Giorgia Bruno, Raffaele Annunziata

**Affiliations:** a Anesthesia, Emergency and Burn Intensive Care Unit, “AORN A. Cardarelli”, Naples, Italy; b Anesthesia and Intensive Care Unit, PO “Santa Maria della Misericordia”, Sorrento, Italy

**Keywords:** GI endoscopy, Deep sedation, Anesthesiology, Non-operating room anesthesia (NORA), Airways management

## Abstract

**Introduction:**

Emergency upper gastrointestinal (GI) endoscopy is often prolonged and complex, performed during high-risk conditions. These procedures can be affected by an increased risk for airway compromise. Scarce literature can be found providing guidance on anesthesiological conduct during upper GI endoscopy in Emergency.

**Methods:**

This was a monocentric retrospective study conducted on 96 patients treated in the Emergency Gastroenterology Unit at AORN Cardarelli, between June and October 2023. Key features of moderate and deep sedation procedures were investigated.

**Results:**

There was no statistically significant difference in the incidence of complications and respiratory depression between the patients receiving moderate sedation versus those treated with deep sedation.

**Discussion:**

Adverse outcomes of sedation and analgesia during endoscopic procedures are rare, even during a deep state of sedation.

## 1. Introduction

The term procedural sedation and analgesia (PSA) [1] involves the use of hypnotic and/or analgesic medications to enable performing diagnostic or therapeutic procedures effectively, whilst the patient is closely monitored for potential adverse effect. Overall, there is no consensus on optimal depth of sedation or sedative agents to be used when performing upper GI endoscopy [2,3], however, when it comes to routine, low-risk and moderate-risk endoscopic procedures, the administration of sedatives in unintubated patients, termed “monitored anesthesia care (MAC)”, often obtained using benzodiazepine and/or opioids, is one of the most common sedation methods for GI procedures in North America and Europe. It allows for minimal discomfort to the patients and can be safely administrated without the occurrence of critical desaturation. There is abundant literature consensus stating in can be even performed by trained non-anesthesiologist [4].

The purpose of sedation and analgesia is to relieve patient anxiety and discomfort [5], improve the outcome of the examination, and diminish the patient's memory of the event. Inadequate sedation/analgesia is to be avoided at any cost, given it may result in undue patient discomfort or patient injury because of lack of cooperation.

Drug selection for PSA should be based on ease of reaching and maintaining the desired level of sedation and analgesia, therefore avoiding adverse events caused by excessive dosage or unexpected reactions to the individual drug or drugs combination [8]. As such, the theoretically ideal drug for PSA has a rapid onset, short duration of action and time-independent context-sensitive half-time [9,10]. In addition, it should have a beneficial hemodynamic and respiratory stability profile [11]. As most of the available drugs for PSA do not cover both the hypnotic and analgesic endpoints [12,13], drug combinations are mostly required [14,15].

The present study aims at demonstrating that deep sedation, obtained with the administration of ketamine, propofol, benzodiazepine and opioids, is a safe and viable option when it comes to complex upper GI endoscopy, especially in those patients where the risk-benefit ratio for general anesthesia is not optimal [6,7].

## 2. Methodology

This was a single-center observational retrospective study. We completed this study between June and October 2023 at the Emergency Gastroenterology Unit of Hospital Antonio Cardarelli (Naples, Italy). This study was approved by the local institutional review board (Ethics Committee protocol number 00014602 – 07/06/2023) and conducted according to the Declaration of Helsinki.

We established inclusion/exclusion criteria for the study as follows:

All patients >18 years of age undergoing emergency upper GI endoscopy were enrolled in this study. Patients were excluded if they met any of the following criteria: GCS <12, patients intubated needing mechanical ventilation, acute heart failure, contraindications or allergies to the drugs used and pregnant or breastfeeding women.
The aim of this study was to evaluate the frequency of complications during emergency GI endoscopies under sedation. In particular, our primary endpoint was comparing the number of significant desaturations between Group 1 (Deep sedation, ASA level 3) and Group 2 (moderate sedation, ASA level 2).
Secondary outcomes were to evaluate the incidence of other adverse effects (bradycardia, bradypnea, hypotension and need for orotracheal intubation for airway protection).

### 2.1. Data collection and procedure

Demographic information, comorbidities, and vital signs were recorded in the standard data registry form from the hospital's computerized medical record.

Concerning the procedure, vital signs of all patients were monitored (e.g. pulse oximetry, heart rate (HR) and non-invasive blood pressure). After venous line placement, supplemental oxygen therapy with nasal cannula 4–6 l/min was started. Induction and maintenance of sedation was performed administering a combination of propofol and midazolam and/or ketamine and/or fentanyl.

At the end of the endoscopy, flumazenil 1 mg as a single dose was given to the patients before being transferred to the recovery room. In the recovery room, vital parameters and Aldrete score were recorded [20].

Complications, such as important desaturation (SpO2<90 %), bradycardia, bradypnea or orotracheal intubation were recorded.

Lastly, we monitored the elapsed time of each EGDS, in order to see if deep sedation could allow the endoscopist to quicker complete the procedure. In this study, the Richmond Agitation–Sedation Scale (RASS) has been used to describe the patient's level of consciousness. RASS is a 10-point scale, with four levels of anxiety or agitation (+1 to +4 [combative]), one level to denote a calm and alert state (0), and 5 levels of sedation (−1 to −5) culminating in unarousable (−5). Furthermore, the American society of anesthesiologist (ASA) was used to defined four levels of sedation, where level 4 corresponds to general anesthesia.

### 2.2. Group allocation

Being a retrospective study, we collected data of all the patients that underwent emergency upper GI and, based on the level of sedation they received, we could define two groups:

Group 1 was composed by all the patients that underwent emergency upper GI under deep sedation (ASA level 3, according to the American Society of Anesthesiologist).
Group 2 was composed by all the patients that underwent emergency upper GI under moderate sedation (ASA level 2, according to the American Society of Anesthesiologist).

### 2.3. Statistics

Categorical data were summarized with counts and frequencies, compared using contingency tables, Chi-square test. Distribution of the continuous variables was evaluated with Shapiro Wilk test and summarized using median and Interquartile range (IQR), compared using Wilcoxon rank sum test. Comparisons of primary endpoint were performed using Odds Ratio (OR) test and 95 % confidence interval. Type 1 error was accepted as 5 %. R statistical software was used for the statistical analyses and production of graphs.

## 3. Results

In our analysis, we included 96 patients who fulfilled inclusion criteria, allocating them in one of the two groups: group 1 (n = 54 – DEEP), patients with a deep level of sedation during the procedure (ASA level 3); group 2 (n = 42 – MOD), patients with a moderate level of sedation during the procedure (ASA level 2).

Demographic and clinical characteristics are reported in Table 1, showing the two groups are homogenous for comparative analysis. In addition, there were no significant differences in ASA Physical Status Classification System (ASA score) between the two groups.

**Fig. 1:**
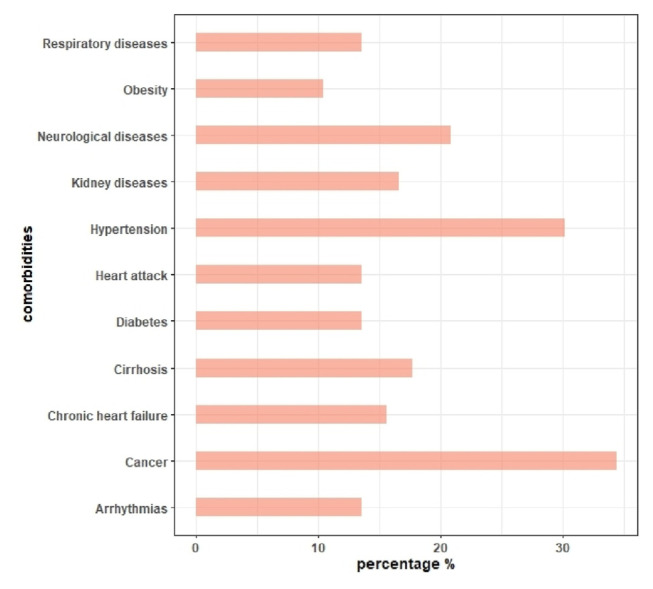
Distribution of the main comorbidities (%).

Comorbidities are reported in Fig. 1. Our data shows that most are cancer patients (34,4 %, such as gastric, colon, pancreatic cancer) or suffering from cardiovascular diseases (30.2 % hypertension, 13.5 % heart attack, 15.6 % heart failure, 13.5 % arrhythmias). Other comorbidities are distributed as follows: metabolic diseases (10.4 % obesity, 13.5 % diabetes, 17.7 % cirrhosis), kidney diseases (16.7 %), respiratory diseases (13.5 %, asthma, COPD) and neurological diseases (20.8 %, such as stroke, dementia).

The main indications for performing an emergency endoscopic procedure were: hematemesis (49 %), melena (30.2 %), food bolus (12.5 %) and foreign bodies (8.3 %).

Duration of the procedures was not significantly different between the two groups.

**Fig. 2:**
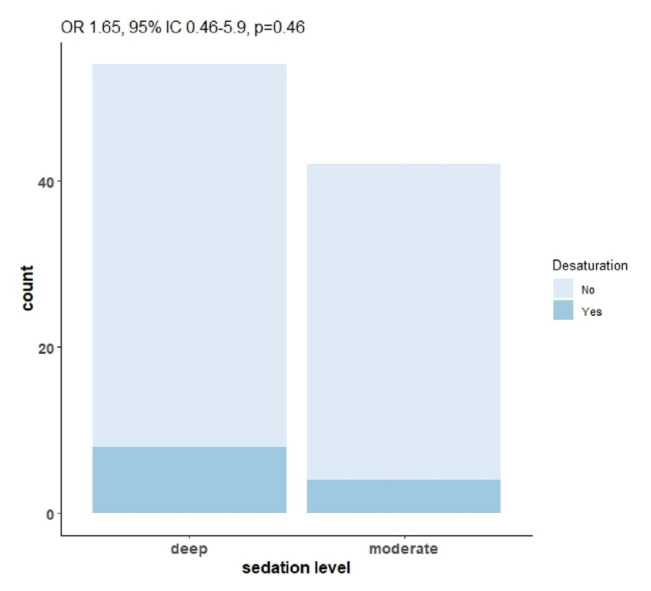
Desaturation occurred in both MOD and DEEP groups. Not statistically significant.

Significant desaturation was detected in 8 patients (14.8 %) in the DEEP group compared to 4 patients (9.5 %) in the MOD group (OR 1.65, 95 % IC 0.46–5.9) Fig. 2. These complications were resolved with jaw thrust, increased oxygen support or with mask ventilation, allowing the procedure to come to an end without further complications. Only 3 patients required Orotracheal Intubation during the procedure. No other adverse events were detected (bradycardia, bradypnea and hypotension).

There were no significant differences in SpO2 at operating room admissions or discharge between the two groups (p-value = 0.13 and p-value = 0.12).

All patients obtained an Aldrete score ≥ 8 at the end of the procedure (indicating that these patients were ready to be discharged from Recovery Room).

## 4. Discussion

With the intrinsic limits of a retrospective study, we intended to demonstrate that deep sedation in emergency upper GI endoscopy performed during high-risk conditions, like acute upper GI hemorrhage, sub-acute bowel obstruction/ileus, food bolus, achalasia, on high-risk patients, i.e. elderly patients, patients with morbid obesity, liver or kidney failure, patients with chronic respiratory diseases, presenting SpO2 < 95 % with supplemental oxygen, is a safe and viable option, with very scarce complications [16–18]. In fact, the ability to independently maintain a ventilatory function [19], the need for assistance in maintaining a patent airway, and cardiovascular function were all investigated in this study.

Our study shows that among the 96 patients treated in the Emergency Gastroenterology Unit at AORN Cardarelli Hospital, only 12 of them underwent a significant desaturation event, quickly resolved with supplemental oxygen and a jaw thrust, with no long-term implications. Furthermore, there were no statistically significant difference of outcome between Group MOD sedation and Group DEEP sedation (p-value = 0.46), meaning deep sedation is in no way riskier or more hazardous when compared to a moderate sedation.

According to Cappellini et al., 2024 [15], whenever feasible, a pre-sedation assessment should be conducted, and it should comprise a focused medical history, ASA score, physical examination with airway evaluation to rapidly identify a potentially difficult airway, a review of comorbidities, medications and allergies, and an inquiry about previous sedation, anesthesia, and surgery history, in order to immediately recognize red flags that could increase the likelihood of adverse events. Abundant literature already agrees on the safety of procedural sedation for elective GI endoscopy [2]. Smally AJ et al. [21] addressed the topic of emergency procedural sedation with propofol, ketamine, midazolam and fentanyl being safe, granted the employment of proper monitoring. In 2016, a meta-analysis performed by Bellolio MF et al. [16] stated that the most common adverse event during procedural sedation in the Emergency Department was hypoxia, with an incidence of 40.2 per 1000 sedations (95 % CI = 32.5 to 47.9), followed by vomiting and hypotension Severe adverse events requiring emergent medical intervention were rare, with one case of aspiration in 2370 sedations (1.2 per 1000), one case of laryngospasm in 883 sedations (4.2 per 1000), and two intubations in 3636 sedations (1.6 per 1000). Our findings are all in accordance with these papers, and confirm that deep sedation proved to be sound even during emergency, life threatening procedures performed on a high-risk population. In addition, as stated in many papers [2,5], there were no statistically significant difference between MOD and DEEP groups in duration of the procedure, or adverse events in the recovery room.

### 4.1. Limitations

Our study has the great limit of being a monocentric study with a modest sample size, further investigation, perhaps randomized controlled trials, should be performed to better define the safety profiles of emergency GI endoscopy under deep sedation.

## 5. Conclusion

Deep sedation in high-risk patients undergoing Emergency upper GI endoscopy involves the use of hypnotic and/or analgesic medications to enable performing diagnostic or therapeutic procedures effectively, while the patient is closely monitored for potential adverse effect. Emergency upper GI endoscopy is often prolonged and complex, performed during high-risk conditions, like acute upper GI hemorrhage, sub-acute bowel obstruction/ileus, food bolus, achalasia, on high-risk patients (i.e. elderly, morbid obesity and patients with chronic respiratory diseases, presenting SpO2 < 95 % with supplemental oxygen). In these cases, deep sedation might give the advantage of a quicker endoscopy and a fully cooperative patient, without exposing them to additional risks.

Our study, with the limit of being a monocentric study with a modest sample size, shows that adverse outcomes of sedation and analgesia during endoscopic procedures are rare and that there are no major differences in adverse outcomes between mildly sedated patients and deeply sedated ones. But most importantly we showed that, even during a deep state of sedation according to the ASA *De*fi*nition of General Anesthesia and levels of Sedation/Analgesia*, there were no severe, life-threatening complications, making this anesthesiological conduct a safe and sound choice, when performed by a trained specialist.

## Figures and Tables

**Table 1:** Demographic and clinical characteristics.

Characteristic	Deep, N = 54^a^	Moderate, N = 42^a^	p-value^b^
**Age (years)**	68 [58, 75]	65 [46, 76]	0.3
**Sex**			0.14
Female	13 (24 %)	16 (38 %)	
Male	41 (76 %)	26 (62 %)	
**Weight (Kg)**	73 [60, 80]	70 [61, 75]	0.4
**ASA**			0.9
1	8 (15 %)	6 (14 %)	
2	9 (17 %)	7 (17 %)	
3	29 (54 %)	25 (60 %)	
4	8 (15 %)	4 (9.5 %)	
**Duration**	20 [15, 28]	17 [10, 29]	0.4

a Median [IQR]; n (%).

b Wilcoxon rank sum test-Pearson's Chi-squared test.
